# Wood Anatomy Reveals High Theoretical Hydraulic Conductivity and Low Resistance to Vessel Implosion in a Cretaceous Fossil Forest from Northern Mexico

**DOI:** 10.1371/journal.pone.0108866

**Published:** 2014-10-03

**Authors:** Hugo I. Martínez-Cabrera, Emilio Estrada-Ruiz

**Affiliations:** 1 Estación Regional del Noroeste, Instituto de Geología, Universidad Nacional Autónoma de México, Hermosillo, México; 2 Laboratorio de Ecología, Departamento de Zoología, Escuela Nacional de Ciencias Biológicas – Instituto Politécnico Nacional, Ciudad de México, México; Berkeley, United States of America

## Abstract

The Olmos Formation (upper Campanian), with over 60 angiosperm leaf morphotypes, is Mexico's richest Cretaceous flora. Paleoclimate leaf physiognomy estimates indicate that the Olmos paleoforest grew under wet and warm conditions, similar to those present in modern tropical rainforests. Leaf surface area, tree size and climate reconstructions suggest that this was a highly productive system. Efficient carbon fixation requires hydraulic efficiency to meet the evaporative demands of the photosynthetic surface, but it comes at the expense of increased risk of drought-induced cavitation. Here we tested the hypothesis that the Olmos paleoforest had high hydraulic efficiency, but was prone to cavitation. We characterized the hydraulic properties of the Olmos paleoforest using theoretical conductivity (*K_s_*), vessel composition (*S*) and vessel fraction (*F*), and measured drought resistance using vessel implosion resistance 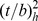 and the water potential at which there is 50% loss of hydraulic conductivity (*P_50_*). We found that the Olmos paleoforest had high hydraulic efficiency, similar to that present in several extant tropical-wet or semi-deciduous forest communities. Remarkably, the fossil flora had the lowest 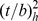, which, together with low median *P_50_* (−1.9 MPa), indicate that the Olmos paleoforest species were extremely vulnerable to drought-induced cavitation. Our findings support paleoclimate inferences from leaf physiognomy and paleoclimatic models suggesting it represented a highly productive wet tropical rainforest. Our results also indicate that the Olmos Formation plants had a large range of water conduction strategies, but more restricted variation in cavitation resistance. These straightforward methods for measuring hydraulic properties, used herein for the first time, can provide useful information on the ecological strategies of paleofloras and on temporal shifts in ecological function of fossil forests chronosequences.

## Introduction

The expression of anatomical and morphological traits is subject to biophysical constraints imposed by environmental demands. Consequently, they can provide important information about ecological strategies of fossil assemblages [1], [2] and paleoclimate (e.g. [3], [4]). Because a large amount of water is needed to maintain plant growth, water is a major factor limiting plant distribution and trait expression. Water loss and carbon fixation are linked because during CO_2_ uptake water is transpired to the atmosphere [5], and because diffusion coefficients for water are larger than for CO_2_, efficient carbon fixation and plant growth requires a disproportionately high hydraulic supply to the leaves to meet evaporative demands during photosynthesis. This relationship between stem hydraulic capacity with tree growth rate [6], [7] and leaf photosynthetic capacity is well established [8].

Across vegetation types and biomes, at low latitudes and altitudes, there is a positive relationship between water availability and xylem conduit size, such that in xeric environments vessel size is smaller on average than in tropical humid environments [9], [10], [11]. Plant water transport efficiency is directly related to the hydraulic conductivity of xylem, which is mostly determined by conduit size [12], [13], [14]. As warm wet environments are also more productive regions, hydraulic efficiency should be directly linked to carbon fixation, as has been empirically confirmed (e.g. [15]). In places with high water availability hydraulic capacity is increased by decreasing resistance to water flow in the xylem (e.g. increasing vessel diameter). In these warm wet environments, plants can maintain high transpiration rates and maximize carbon fixation and growth [16]. However, in dry conditions, wide, hydraulically efficient vessels are also more prone to experience mechanical failure [17], [18], [19] or drought induced cavitation [14], [20], [21].

Although drought induced cavitation occurs along the entire water availability continuum [22], [23], plants from drier regions with smaller vessel diameters are in general more capable to cope with cavitation because it occurs at higher xylem tensions (lower water potential) than in wet adapted plants with larger vessel diameters. The consequence of vessel cavitation is the disruption of the water column, which reduces xylem hydraulic conductivity and the overall plant water supply to the photosynthetic surface [24]. Cavitation can thus be translated into a decrease in photosynthesis [15] and a drop in stomatal conductance [25], which further increase xylem tension and cavitation [24], [26]. Cavitation resistance in extant plants is usually quantified using the water potential at which there is 50% loss of hydraulic conductivity (*P_50_*). As *P_50_* values are impossible to measure in fossil wood samples, we used a metric developed by Hacke et al. [18], the vessel resistance to implosion metric (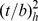, defined below) that can be used to approximate cavitation resistance. 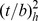 explains between 80% [18] and 95% [19] of *P_50_* variation and is essentially a measure of vessel wall reinforcement. 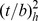 is entirely based on vessel anatomy and therefore can be used to determine drought tolerance thresholds (*P_50_*) in fossil woods.

In this paper we used vessel anatomy to determine key functional traits related to drought resistance (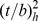 and *P_50_*) and hydraulic capacity (potential conductivity *K_s_*, vessel fraction *F*, and vessel size contribution metric *S*) of a fossil forest from the Olmos Formation (upper Campanian), Coahuila, Mexico. These two sets of functional traits provide information about the hydraulic functional strategies of the fossil forest. Because of their close link to the environment, they also provide hints as to the climate regime in which the fossil plants grew. With more than 80 different leaf morphotypes [27], [28], [29], [30], [31], [32], [33], [34], mainly angiosperms (80%), the Olmos Formation is one of the richest Cretaceous paleoforests from Mexico and south-central Western Interior of North America (WINA). According to leaf physiognomy paleoclimate estimates, the Olmos paleoforest grew under a tropical climate (MAT 20–23°C) with high water availability (1.5 to 3 m) [28]. The Olmos paleoforest was probably more mesic-adapted than other floras from the southern part of North America during this period [28]. Based on these paleoclimate reconstructions and leaf physiognomy, we expected that this paleoforest would have had a very efficient hydraulic system to meet high evaporative demands, but we also expected that this hydraulic efficiency should be paired with a high risk of embolism formation. In other words, in the conduction efficiency-cavitation risk trade-off continuum, the Olmos paleoforest should represent a highly hydraulically efficient and embolism-prone ecological strategy only suitable under warm, wet environments. Moreover, large leaf area and tree heights, estimated to be up to 35 m in some species [28], suggest a highly productive environment and efficient carbon fixation, which would require an efficient hydraulic system.

## Material and Methods

### Geological setting

**Figure 1:**
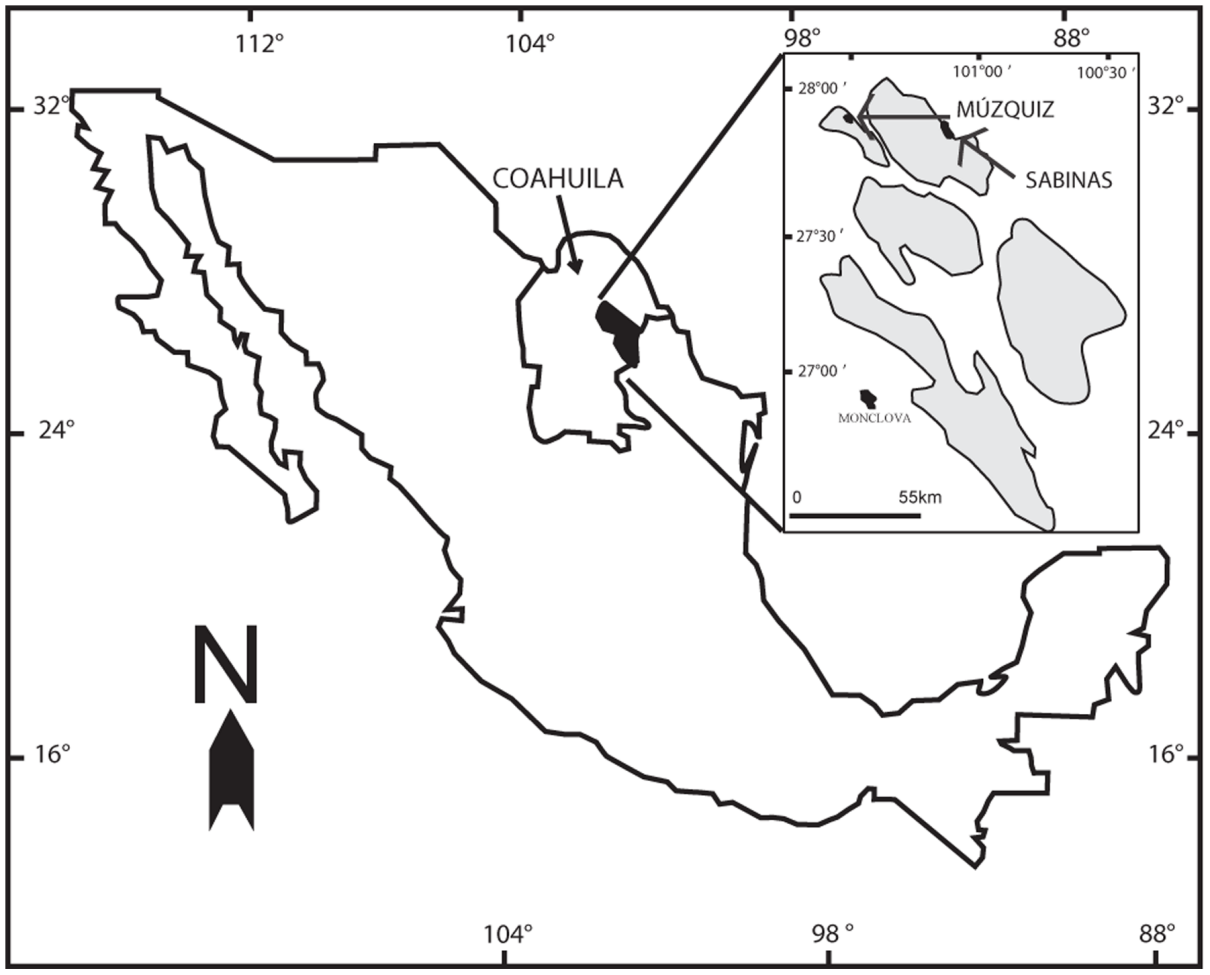
Location map of the Olmos Formation in the Sabinas Basin (in gray). Sampled sites in Sabinas and Múzquiz (Atascoso and Santa Elena Ranchs) are identified by arrows.

The outcrops of the Olmos Formation (upper Campanian) are found in the Sabinas Basin in the state of Coahuila in northern Mexico (Fig. 1) [35], [36], [37], [38]. The Olmos Formation represents a fluvial-deltaic system with four main depositional sub-environments [36] that include: 1) swampy areas with restricted circulation, 2) floodplain environments and/or a lagoon system with open circulation, 3) fluvial environments, likely including braided rivers and 4) meandering rivers [36]. The angiosperm woods, along with numerous dinosaur bones, were collected in environments representing meandering rivers [36]. The woods were not found in growth position and despite the large size of some samples, we assumed that some transport occurred.

All necessary permits were obtained for the described study, which complied with all relevant regulations. These permits were issued by the Instituto Nacional de Antropología e Historia (INAH) and the material is housed at the National Collection of Paleontology (Universidad Nacional Autónoma de México).

### Anatomical measurements

The anatomical characteristics of the 10 wood xylotypes thus far described for the Olmos Formation [27], [39] were measured on transverse sections obtained using a standard thin-section technique. In 11 field trips we have collected nearly 100 samples, from these, we have recognized 10 dicot, 5 palms and 2 gymnosperm (Podocarpaceae and Taxodiaceae) xylotypes. Despite that most of the xylotypes were identified in the first two field visits, and no new xylotypes have been collected in recent visits, we do not discard the possibility of finding new morphospecies given the outstandingly high diversity of the leaf flora. The10 wood xylotypes we studied here represent all the dicot fossil woods collected so far and therefore, despite their relatively low number, they are a good representation of the dicot flora. We measured more than one sample for six of the ten species/xylotypes (i.e., *Coahuiloxylon terrazasiae*, *Javelinoxylon* xylotype 2, *Javelinoxylon weberi*, *Metcalfeoxylon* xylotype 1, *Muzquizoxylon porrasii* and *Wheeleroxylon atascosense*). See Table 1 for species means and standard deviations, and Table S1 and Table S2 for values of each measured vessel and means per sample, respectively. Species means for each of the measured functional traits were calculated based on the mean of the each of the samples per xylotype.

**Table 1:** Means and standard deviations of the hydraulic and drought resistance traits for the Olmos Formation species/xylotypes.

Species	*Affinities*	*K_s_*	*F*	*S*	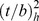	*P_50_*	N
*Coahuiloxylon terrazasiae*	Anacardiaceae/Burseraceae	4.28 (2.31)	0.22 (0.55)	0.00168 (0.0005)	0.0038 (0.0014)	−1.25 (0.22)	2
*Javelinoxylon xylotype 1*	Malvaceae	12.62	0.33	0.0045	0.0103	−2.26	1
*Javelinoxylon xylotype 2*	Malvaceae	1.63 (0.22)	0.16 (0.011)	0.00069 (0.00004)	0.0346 (0.0065)	−6.01 (1.005)	2
*Javelinoxylon weberi*	Malvaceae	6.97 (2.24)	0.36 (0.72)	0.0009 (0.00005)	0.0106 (0.0059)	−2.30 (0.091)	2
*Metcalfeoxylon xylotype 1*	Incertae sedis	5.8 (0.93)	0.15 (0.012)	0.0095 (0.0007)	0.0106 (0.0028)	−2.3 (0.043)	3
*Muzquizoxylon porrasii*	Cornaceae	0.92 (0.45)	0.17 (0.065)	0.00016 (0.00002)	0.064 (0.0005)	−10.58 (0.084)	2
*Olmosoxylon upchurchii*	Lauraceae	54.46	0.68	0.0094	0.00159	−0.909	1
*Quercinium centenoae*	Fagaceae	69.82	0.65	0.0175	0.00101	−0.82	1
*Sabinoxylon pasac*	Ericales	30.81	0.43	0.0116	0.00303	−1.13	1
*Wheeleroxylon atascosense*	Malvaceae	38.94 (3.94)	0.66 (0.033)	0.00524 (0.00026)	0.00607 (0.0004)	−1.6 (0.064)	2

*K_s_* =  potential conductivity; *F* =  vessel fraction; *S* =  vessel size to number ratio; 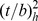 =  vessel implosion resistance; *P_50_* =  cavitation resistance; N  =  number of studied samples per xylotype.

To determine potential conductivity (*K_s_*), vessel resistance to vessel implosion 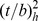, and *S* and *F* metrics, we calculated dimensions of 62 to 398 vessels per species (See Table S1 for the number of vessels measured per sample and species/xylotype). The number of vessels measured varied as a function of their density. To calculate vessel dimensions we first randomly selected radial sectors (cross sectional area limited by rays) and measured all the vessels contained in the sector. For species with a limited number of vessels (low vessel density) such as *Quecinium centenoae* and *Metcalfeoxylon* xylotype 1, we measured all the vessels that the preservation allowed. Vessel outlines were drawn using a graphics tablet (Intuous 3, Wacom, Kita Saitama-Gun, Saitama, Japan). Vessel area was calculated using XTools of ArcView (version 3.2, ESRI, Redlands, CA, USA). For details of the measuring protocol, see Martínez-Cabrera et al. [40]. We calculated vessel lumen diameter, including mean vessel diameter (*d_mean_*) and hydraulic mean (*d_h_*), using diameters of circles with the same area as the individual vessel lumens, following Kolb and Sperry [22]. *d_h_* was calculated as the sum of the contribution of all conduit diameters (*∑d^5^*) divided by the total number of vessels (*∑d^4^*) [22], [41]. *d_mean_* was then used, together with other variables (see below), to calculate potential conductivity, while *d_h_* was used for estimating the squared vessel-wall thickness-to-span ratio 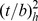
_,_ a proxy for the resistance to vessel implosion.

We calculated potential conductivity per stem cross sectional area (*K_s_*) following Zanne et al. [13]: 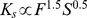, where *F* is the vessel fraction and *S* is a vessel size contribution metric. Vessel fraction *F* is mean vessel area 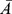 times vessel density (*N*) (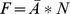; mm^2^·mm^−2^), and *S* is the ratio between the same anatomical traits (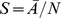; mm^4^). 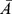 is the mean individual vessel cross sectional area and *N* is the vessel number per unit of sapwood area [13]. Vessel fraction *F* approximates the fraction of cross sectional area occupied by vessel space [13], [42] and *S* measures the variation in vessel size composition. Higher values of *S* indicate a greater contribution of wide vessels to water conduction in a given area [13].

The safety factor for vessel implosion [18] was calculated using the squared vessel-wall thickness-to-span ratio 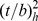, where the paired vessel wall thickness, *t* , is the thickness of the wall between two adjacent vessels and *b* is the diameter of the conduit closest to the hydraulic mean diameter (*d_h_*). The 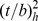 was measured in 16 to 58 vessel pairs per sample, in which at least one of the vessel in a pair was within ±5 µm of the hydraulic mean diameter (*d_h_*). The number of vessels used for each sample and species/xylotype is presented in Table S2 and Table S3. *b* has a strong influence on 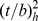; therefore, we do not expect much variation between vessels. The relatively small sample size for some species is because of the insufficient number of vessel pairs within the desired vessel diameter. We also measured implosion resistance in species with solitary vessels that had conducting cells other than vessels (e.g. vascular and vasicentric tracheids) associated with them, as was the case for *Sabinoxylon pasac*, *Quercinium centenoae*, and *Metcalfeoxylon* xylotype 1. Since conduit 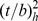 captures between 80 and 95% of *P_50_* variation, we used the relationship between these two variables to estimate cavitation resistance of the fossils (*P_50_* = −0.662–154.646 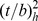; regression equation was kindly provided by Uwe Hacke, University of Alberta).

### Comparison with extant communities

The values of the characteristics measured are a good proxy for the hydraulic function of the individual/species studied, in this sense they are by themselves measurements, not predictions of a value (as are the paleoclimate estimates using plant structures, which require a minimum number of species). We compared the calculated *K_s_*, 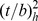, *F* and *S* values from the Olmos Formation woods with those from nine extant communities. All the species analyzed in these extant communities were dicots, and the anatomical information was taken from previous studies [1], [40], [43], [44]. These extant communities included a tropical rain forest, a dry deciduous forest and a montane forest from Mexico [1], [43] and several communities from sites with a wide range of precipitation in North and South America [40], [44]. These last extant communities varied from hardwood forest to desert scrub. For this comparison, *K_s_, S* and *F* were calculated for the entire set of extant communities. The lack of information necessary to calculate resistance to vessel implosion in the Mexican communities (tropical rain forest, Veracruz; montane forest, Estado de México; dry deciduous forest, Jalisco) restricted our comparisons for this metric to a smaller (6) set of extant localities. Because *K_s_*, and *S* had non-homogeneous error variance term (Levene's test , P<0.0001 in both cases) with regard to vegetation type (i.e. variance of the error term is not constant throughout vegetation types) we used Kruskal-Wallis test to detect overall difference among groups. Then, to detect pairwise differences between the Olmos paleoforest and the extant communities, we performed Mann-Whitney U test. The homoscedastic variables (*F*, implosion resistance and *P_50_*) were compared using ANOVA. Similarly, we ran a Levene's test to assess the homoscedasticity of the error term of the functional traits at different MAP levels. The relationship of those traits with homogeneous variance and MAP (*F* and *S*) was then analyzed, prior a log transformation, using simple linear regression. For those traits with non-homogeneous variance (implosion resistance and *K_s_*,) we ran weighed least squares regressions using variance at each MAP level as weights to fit the model.

Additionally, we used PCA to visualize 1) hydraulic properties (*K_s_, S* and *F*) of the Olmos Formation woods with the full extant data set and 2) these same hydraulic properties plus implosion resistance in the smaller subset of communities. To determine whether vegetation type/communities were well discriminated by their hydraulic properties we carried out a between-group PCA and assessed the significance of the results using a Monte-Carlo permutation test on the between group inertia percentage [45], [46]. Significance was calculated by comparing the between-group observed differences with the distribution of 999 permutations simulated. We used the R package ade4 [47], [48] to perform this analysis.

## Results

### Potential hydraulic conductivity

**Figure 2:**
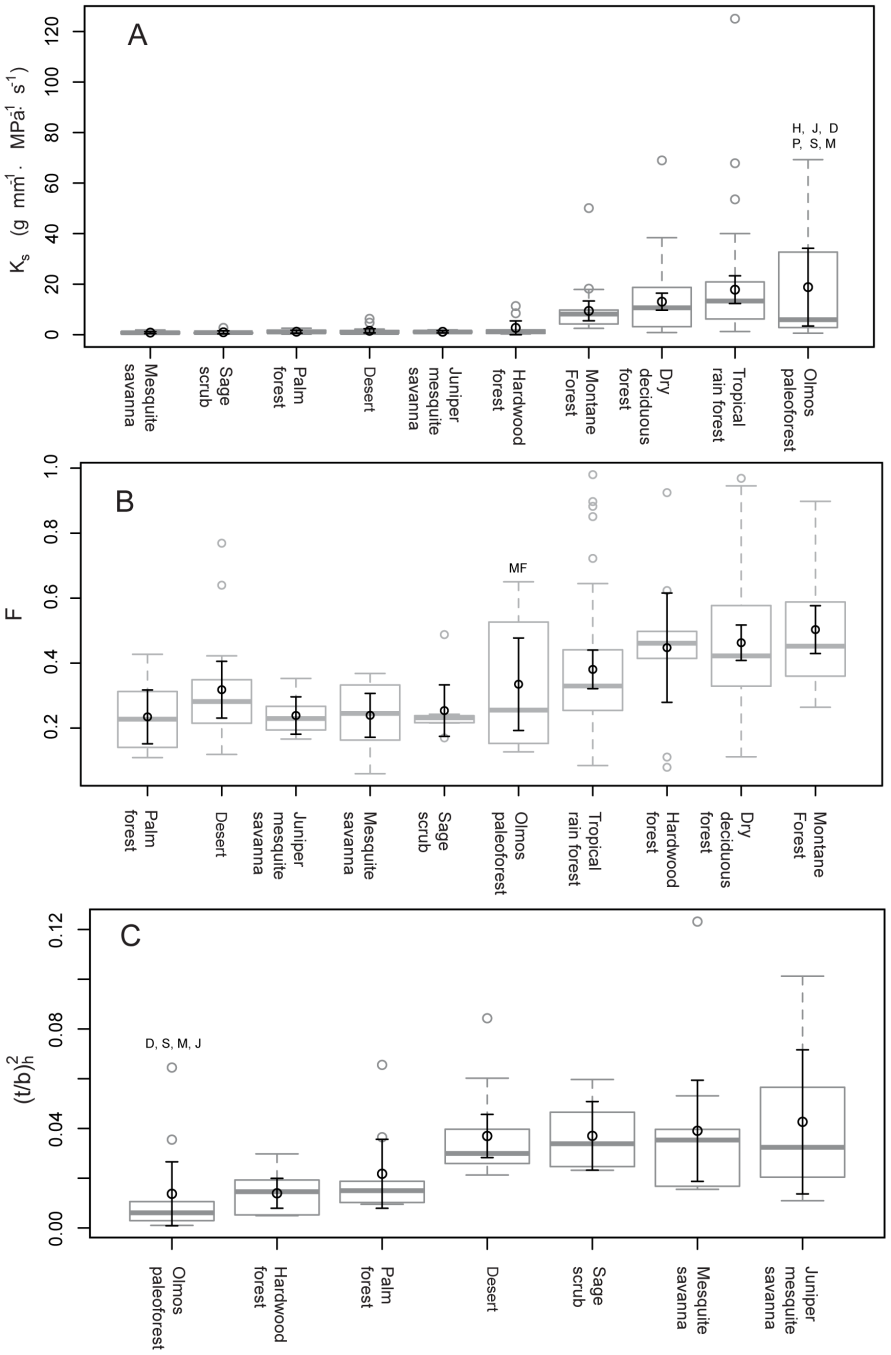
Comparison of a) potential conductivity, b) vessel fraction and c) implosion resistance between the Olmos Formation fossil woods and extant communities. Box plots in grey show the median and interquartile distance of each one of the variables. The black circle and error bars represent the mean and ±95% confidence intervals. Box plots showing implosion resistance only includes the drier extant communities and the Olmos formation flora. Sample sizes: mesquite savanna (M) = 11, sage scrub (S) = 8, palm forest (P) = 9, desert (D) = 17, juniper-mesquite savanna (J) = 7, hardwood forest (H) = 10, montane forest (MF) = 25, dry deciduous forest = 56, tropical rain forest  = 54, Olmos flora = 10. The two different desert communities were pooled for this analysis. Significant difference between the Olmos pleoflora and extant vegetation types were determined using Mann-Whitney test for potential conductivity, and ANOVA for *F* and implosion resistance.

There were significant differences of conduction capacities among all communities (Kruskal-Wallis chi squared = 106.3237, p<0.001). Although the Olmos paleoforest had the highest mean potential conductivity per stem cross sectional area (*K_s_* = 22.6 g·mm^−1^·Mpa^−1^·s^−1^), it was not significantly different from the hydraulic conductivity of the tropical rain forest (Mann-Whitney Z = 265, p = 0.93; *K_s_* = 17.81 g·mm^−1^·Mpa^−1^·s^−1^), the montane forest (Mann-Whitney Z = 103, p = 0.43; *K_s_* = 9.41 g·mm^−1^·Mpa^−1^·s^−1^) and the dry deciduous forest (Mann-Whitney Z = 317, p = 0.51; *K_s_* = 3.06 g·mm^−1^·Mpa^−1^·s^−1^) (Fig. 2a). The Olmos paleoforest had significantly higher *K_s_* than the dry the North American hardwood forest (Mann-Whitney Z = 14, p = 0.005; *K_s_* = 2.71 g·mm^−1^·Mpa^−1^·s^−1^) and the remaining extant communities (Fig. 2a). The South American mesquite savanna had the lowest theoretical hydraulic capacity (*K_s_* = 0.28 g·mm^−1^·Mpa^−1^·s^−1^). Although the Olmos paleoforest had a high spread in *K_s_*, ranging from 0.9 (g·mm^−1^·Mpa^−1^·s^−1^) in *Muzquizoxylon* to 69.8 (g·mm^−1^·Mpa^−1^·s^−1^) in *Quercinium* (Table 1), the tropical rain forest from Los Tuxtlas, Veracruz had a larger range in values.

### Vessel composition and vessel fraction

Patterns of vessel composition *S* (not shown) were parallel to those for *K_s_*. There was an overall difference in *S* values across the studied communities (Kruskal-Wallis chi squared = 124.5, p<0.001). *S* values of the tropical rainforest and the Olmos paleoforest were not significantly different (Mann-Whitney Z = 302, p = 0.62; 0.006 mm^4^ vs. 0.0054 mm^4^). In these two communities, higher *S* indicates that fewer larger vessels have greater hydraulic contribution. The *S* metric was significantly lower in the dry-deciduous forest (Mann-Whitney Z = 435, p = 0.008; 0.0034 mm^4^) and the in remaining of the extant communities. The montane forest (0.0014 mm^4^), the juniper/mesquite savanna (0.00013 mm^4^), North American hardwood forest (0.000119 mm^4^), palm forest (0.000114 mm^4^), desert (7.6e-05 mm^4^), mesquite savanna (5.3e-05 mm^4^) and sage scrub (4.9e-05 mm^4^) had *S* values several orders of magnitude lower than the Olmos paleoforest, indicating that numerous small vessels comprise the conducting area in the woods from these communities.

We detected an overall difference among vegetation types in *F* values (ANOVA, F_9, 198_ = 4.5, p<0.001). Vessel lumen fraction (*F*) was largest in the montane forest, where more than 50% (0.5 mm^2^·mm^−2^) of the cross sectional area is occupied by vessels, followed by the dry-deciduous forest (0.46 mm^2^·mm^−2^), hardwood forest (0.44 mm^2^·mm^−2^), and tropical rainforest (0.38 mm^2^·mm^−2^). The proportion of cross sectional area occupied by vessels in the Olmos paleoforest is around 38%, while drier communities had lower *F* values, ranging from 0.31 in the desert vegetation to 0.23 in palm forest, mesquite savanna and juniper mesquite savanna (Fig. 2b). The Olmos paleoforest was only significantly different from the montane forest (F_1, 34_ = 5.88, p = 0.02).

### The safety factor for vessel implosion

There was a significant difference in resistance to implosion 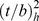 among the compared vegetation types (ANOVA, F_6,65_ = 3.59, p = 0.0038). Vessel implosion resistance was the lowest in the Olmos paleoforest (conduit 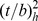 = 0.0145) and ranged from 0.001 in *Quercinium* to 0.064 in *Muzquizoxylon* (Table 1). *Muzquizoxylon* drove mean paleocommunity conduit 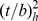 to higher values (Fig. 2c). As a result, the Olmos paleoforest was not significantly different (F_1,19_ = 0.0027, p = 0.95) from the North American hardwood forest and Argentinean palm forest (F_1,19_ = 0.95, p = 0.34) with a conduit 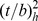 = 0.0139 and 0.021, respectively. If we compare the medians, instead of the means, the vessel implosion thresholds of the fossil assemblage (0.0082) are lower to those of the hard wood (0.0145) and palm forests (0.0149). The desert (F_1,26_ = 11.6, p = 0.002) and the remaining vegetation types had significantly higher vessel implosion resistance than the Olmos paleoforest.

**Figure 3:**
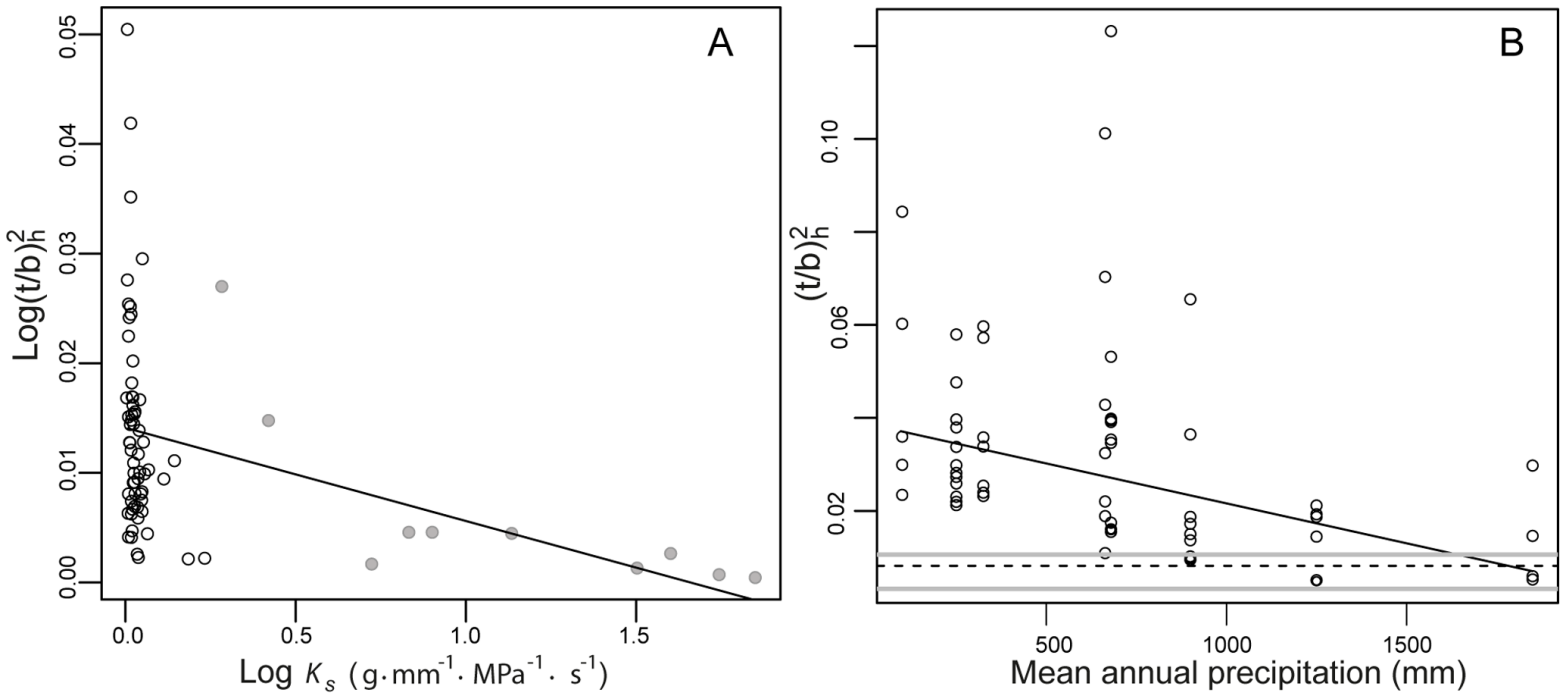
Implosion resistance as a function of a) potential conductivity, b) MAP. In figure 3a the Olmos formation xylotypes are in grey; black circles are the extant communities presented in Figure 2c. In figure 3b the horizontal lines show the median (dashed) and first and third quartiles (grey lines) of implosion resistance for the Olmos Formation, the circles are the species of all extant communities in Figure 2c. Regression line in 3a was fitted using simple linear regression on the log-transformed variables, while in 3b using weighed least square regression.

The estimated *P_50_* values in the fossil assemblage, calculated with the regression equation describing its relationship with 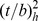, ranged from -0.082 MPa in *Quercinium* to −6 and −10.58 Mpa in *Javelinoxylon* xylotype 2 and *Muzquizoxylon*, respectively (Table 1). The mean paleoforest *P_50_* (−2.9 Mpa, 95% CI = −4.82, −1.01) was driven to higher values by these two species. The median *P_50_* of the fossil assemblage was −1.9 Mpa. We found a significant trade-off between *K_s_* and resistance to vessel implosion (R^2^ = 0.14, p<0.001; y = 0.014–0.008x), indicating that the very high hydraulic capacity of the Olmos fossils comes at the expense of high susceptibility to vessel implosion/cavitation (Fig. 3a). Low *P_50_* values indicate a high risk of cavitation at relatively low water stress (Table 1).

In the extant species' data set, mean annual precipitation (MAP) was significantly related with resistance to vessel implosion (R^2^ = 0.32, p<0.001; y = 3.8–1.72x, Fig. 3b). None of the vessel conduction metrics *F* (R^2^ = 0.02, p = 0.27; y = 0.025+2.2e–5x), *S* (R^2^ = 0.003, p = 0.67; y = 1.19–5.5e–5) or *K_s_* (R^2^ = 0.01, p = 0.78; y = 5.6–4.19x) was significantly associated with MAP, suggesting that resistance to implosion has a greater potential to infer precipitation than any other metric describing water conduction properties. Assuming that resistance to vessel implosion can provide at least a rough approximation of water availability and based on the lines in Figure 3b representing median and the first and third quartiles, we suggest that precipitation in the Olmos paleoforest was comparable to that of our wettest extant sites.

### Between-PCA analysis

**Figure 4:**
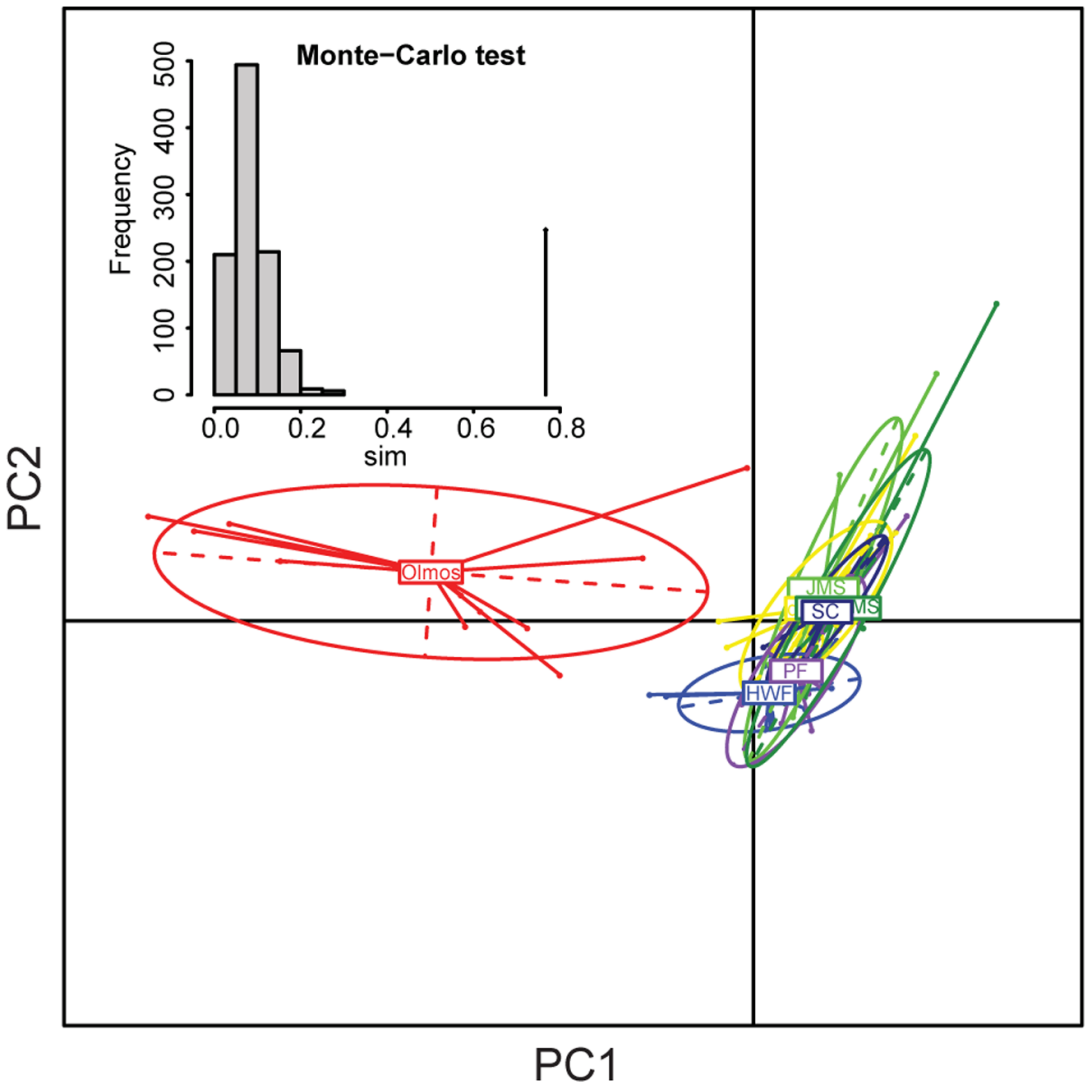
PCA plot portraying hydraulic strategies among communities. Between-Class principal component analysis showing ellipses and gravity centers for extant communities and the Olmos paleoforest. Wet tropical, dry deciduous and montane forest were not included in this analysis. The insert in the upper left corner is the histogram of 1000 simulated values for the between-groups PCA and the observed value (vertical line at sim = 0.78). Sim  =  ratio of between class and total inertia. HWF =  hardwood forest, JMS =  juniper-mesquite savanna, MS = mesquite savanna, PF =  palm forest, SC =  sage scrub.

**Figure 5:**
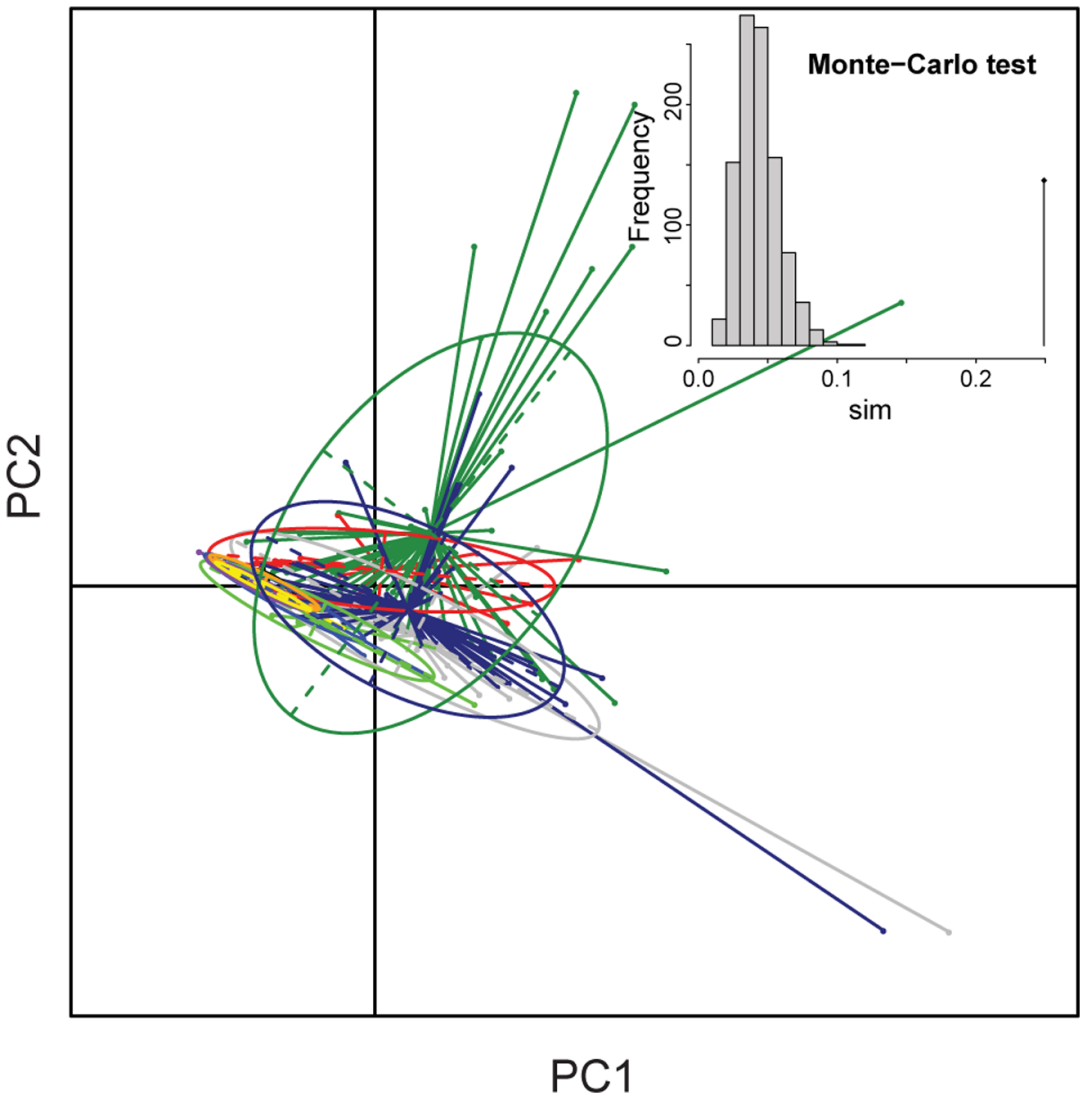
PCA plot portraying hydraulic strategies among all the studied communities. Between-Class principal component analysis shows the ellipses and gravity centers. This plot is based on only hydraulic traits (*F*, *S* and *K_s_*). The insert in the upper right corner is the histogram of 1000 simulated values for the between-groups PCA and the observed value (vertical line at sim = 0.25). Sim  =  ratio of between class and total inertia. Red = Olmos paleoforest, dark green  =  tropical rain forest, light green  =  hardwood forest, light yellow  =  sage scrub, dark yellow =  palm forest, light blue  =  desert, dark blue =  dry deciduous forest, grey  =  montane forest, black =  juniper mesquite savanna, purple =  mesquite savanna.

Species from the Olmos Formation occupied a different region of the functional space in the PCA analysis including hydraulic properties (*K_s_, S* and *F*) and implosion resistance (Fig. 4). The three first PCA axes explained 99% of the variation. In this analysis, hydraulic capacity and implosion resistance are orthogonal (PC loadings for both PCA analysis are presented in Table S4). In the first PCA axis, *K_s_* had the highest loading, while implosion resistance was highest in the second, and *S* and *F* in the third. For instance, in the PCA plot the Olmos paleoforest represents the hydraulically efficient highly prone to vessel implosion extreme. The PCA analysis also revealed that the Olmos paleoforest had greater variation along the hydraulic efficiency axis relative to the implosion resistance axis (the ellipse of the Olmos flora is elongated parallel to the efficient conduction axis). This pattern suggests that the Olmos paleoforest had a large variety of hydraulic strategies but a more constrained number of strategies in implosion/cavitation resistance. The drier communities showed the opposite pattern, with a large breadth in cavitation resistance variation (and also higher values) but highly constrained hydraulic capacity (with low absolute values) (see positive association between axes in Fig. 4). The Monte Carlo permutation test of the ratio of between-class and total inertia in the between PCA analysis supports the discrimination of these groups (ratio  = 0.78, P<0.001; Fig. 4). In the second PCA analysis (only *K_s_, S* and *F*; for all 9 extant communities and the Olmos forest), where the first two principal components explained 93% of the variation, significant differences in functional space among communities are also supported (ratio = 0.25, P<0.001; Fig. 5). In this analysis it is clear that the tropical rain forest exhibits a larger variation in hydraulic strategies than the rest of the communities, including the Olmos paleoforest. In addition, this second analysis reveals a positive relationship between the first two first axes in the tropical rain forest (high *K_s_* is correlated with high *S*: large conduction capacity is reached with large vessels, see the ellipse orientation in Figure 5) while in drier communities the relationship between these two is negative (large *K_s_*, although overall low in most of them, is reached by having many small vessels; low S). The Olmos paleoforest did not have any strong pattern in this regard.

## Discussion

In this paper we set out to determine key properties of the hydraulic system of the Olmos Formation woods. Traits related to hydraulic capacity and vulnerability are important because, as biophysical constraints link them to environmental variation, they provide direct information on ecological strategies of fossil assemblages. For instance, given the warm, wet environment estimated for the Olmos Formation from leaves [28], we expected a highly efficient, highly prone to cavitation hydraulic system. Indeed, our analyses suggest that hydraulic capacity and vulnerability to cavitation at low water stress were high in the Olmos Formation fossil woods.

### Hydraulic capacity of The Olmos Formation

The hydraulic capacity of the Olmos paleoforest was similar to several extant communities including wet tropical, dry deciduous, or montane forest. This suggests that the use of *K_s_* in fossil woods provides information allowing characterization of conduction efficiency differences among communities, but that similar conduction efficiencies can be found under relatively different precipitation regimes. For instance, *K_s_*, has enough power to detect differences in conduction capability between dry and wet floras and therefore may provide only rough estimates of water availability in paleoforests. This is further supported by the absence of a significant relationship between *K_s_* and MAP in our analysis of extant communities. These two variables have been found to be independent in evergreen angiosperms [49]. There is, however, a significant inverse relationship (instead of positive as would be expected) between these two variables in deciduous angiosperms that has been interpreted as the adaptation to water limitation in plant with this ecological strategy [49]. The inverse relationship observed in that study is because most of the deciduous species analyzed [49] are winter deciduous, and thus, a product of the decrease in vessel size associated with higher thresholds to freezing induced cavitation of small vessels (e.g., [50], [51]). It is also possible that *K_s_* is maximized in seasonally dry habitats (e.g., tropical deciduous forest) because of the need for fast growth and carbon fixation during restricted periods of water availability [49], [52]. In the context of our study, the absence of ring porous species and the faint growth rings in the Olmos Formation species indicate that the flora was not subject to sharp seasonal fluctuations of water transport and growth. Instead, high hydraulic efficiency of the woods analyzed, along with constancy of vessel diameter across growth rings, suggests environmental conditions allowing constant hydraulic capacity throughout the year.

As expected, variation in *S* and *F* yielded similar results. However, *S* and *F* describe different functional aspects of water conduction [13]. Low *S* indicates the presence of many small vessels, which are selective under conditions promoting freezing-induced embolism [13], [16]. Thus the large *S* value of the Olmos woods, which was similar to the S value of the tropical rainforest, suggests the absence of freezing temperatures. *F*, on the other hand, is related to potential conductivity, but also describes the amount of cross sectional space occupied by lumen and, by extension, non-lumen (cell wall) area. This metric is bounded at higher values by mechanical support needs and at lower values by hydraulic requirements [13], [42]. All else being equal, high *F* would indicate low construction cost because growth is achieved by greater gas fraction represented by vessel space [12], [13], [53]. The *F* value of the Olmos paleoforest, which is not the highest in our study (around 0.38 mm^2^·mm^−2^ compared to up to 0.5 and 0.46 mm^2^·mm^−2^ in the montane and dry deciduous forest), suggests that construction cost was high relative to hydraulic efficiency. It is important to keep in mind, however, that the amount of variation of wood density (a proxy for construction cost) explained by vessel fraction or other vessel traits is low [13], [19], [40] and that density is mainly driven by fiber traits (e.g. [40]). High wood fraction is positively related with survival and slow growth rate in shade tolerant species, while increased gas fraction is related to high light requirements and adult stature in fast growing species [12]. In sum, our analysis indicate that the Olmos paleoforest had high hydraulic capacity (high *K_s_*) carried out by few very efficient vessels (high *S*), but the amount of wood committed to water conduction was not very large (*F*).

### Resistance to implosion and cavitation

Given the low estimated cavitation resistance (mean *P_50_* = −2.9 MPa) and vessel wall reinforcement metric (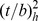 = 0.0145) of the Olmos Formation plants, it seems that, on average, they were at high risk of cavitation even at high water potential (low water stress). This pattern is even more evident if the median *P_50_* (−1.9 MPa) or the mean without outliers (−1.57 MPa) is considered. In a large study considering 230 observations belonging to 167 species from several vegetation types, Maherali et al. [49] showed that the median *P_50_* for extant tropical rain forest was around −1 MPa (n = 41), and ranged from very close to 0 MPa to a little over −6 MPa. Therefore, despite most of species from tropical rain forests being very prone to experience cavitation at high water potential, this biome also has some species that can be quite resistant to drought-induced cavitation. Maherali et al. [49] also found that despite its adaptive significance, *P_50_* exhibited large variation both within and across climates. In some species from the Olmos paleoforest such as *Quercinium* and *Olmosoxylon*, with 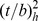 values in the order of 0.001 and estimated *P_50_* of −0.82 and −0.91 MPa, respectively, it is likely that mild water stress would have driven embolism formation and/or vessel collapse. Median *P_50_* for tropical dry forest in the study of Maherali et al. [49] was almost 1 MPa lower than our median (around −2.5 MPa, n = 19), but some species reached over −10 MPa. Of the Olmos Formation woods, only *Muzquizoxylon* reached such low values. It could be argued that the reason behind the extremely high 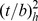 and *P_50_* values in *Muzquizoxylon* is deficiency in preservation, but our observations do not support this conclusion. As relatively high values of cavitation resistance are not absent from extant tropical communities, the high inferred cavitation resistance of *Muzquizoxylon* could also be attributed to intra-community variation.

We showed that there is a significant relationship between 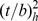 and MAP in extant communities and that the wall reinforcement parameter of the Olmos species were in general within the ranges of extant vegetations with higher precipitation. It has been found that *P_50_* decreases (becomes more negative) with decreasing precipitation in extant evergreen angiosperms and conifers [49]. High resistance to embolism (more negative *P_50_*) has therefore evolved as a strategy to cope with drought. It seems that the utility of 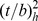 and *P_50_* in detecting water stress in fossil communities is higher than that for *K_s_*, and the calculated values of these metrics for the Olmos paleoforest indicate high water availability.

### Paleoclimate and vegetation

The high hydraulic capacity and low resistance to drought we calculated here reinforces evidence from foliar physiognomy indicating a wet warm climate for the Olmos paleoforest. Around 72% of the species are entire-margined, and 50% of the species with preserved leaf apices have drip tips [28]. Foliar physiognomy estimates a MAT of 20–23°C and a MAP of 1.5–3 m and growing season precipitation of 2 m. In addition, the occurrence of palms and the absence of growth rings in the dicot woods indicate cold month mean temperature >5°C [54]. Our results also agree with the current understanding of the climate regime of the southern WINA during the Late Cretaceous as having tropical temperatures (MAT 18–25°C) and above-freezing annual minimum temperatures [28], [55], [56]. Tropical rainforest is defined by a combination of climatic parameters and plant physiognomical features [57]. These include a MAP of over 1.8 m year^−1^, high MAT (>18°C), a seasonal variation in temperature of less than 7°C and high percent of species with large, entire margined leaves and dip tips [57]. The climate calculated for the Olmos Formation and the physiognomic characteristics of the leaves suggest it was a tropical rainforest. Paleoclimate models show that the frost line during the Maastrichtian was well to the north [58], and based on these simulations [58], Lomax et al. [59] predicted high net primary productivity and leaf area index for the Olmos Formation region. Significantly, the leaf flora of the Olmos Formation also indicates greater precipitation levels than other North American and Western Interior floras during the Late Cretaceous. Based on the small leaf size and low prevalence of drip tips in the paleofloras of the region, Wolfe and Upchurch [56] suggest a subhumid climate throughout the late Cretaceous. Other calculations, based on Climate Multivariate Leaf Analysis Program (CLAMP), support those estimates and propose that in the southern Western Interior precipitation reached up to 1.5 m but it could have been less than 1 m [60], [61]. These estimates contrast with the more mesic conditions of the Olmos Formation flora, with up to 3 m of precipitation, and high prevalence of drip tips. In this sense, the Olmos paleoforest, is physiognomically closer to younger (Paleocene) southern Western Interior floras, where larger leaves, with up to 50% of them having drip tips, are present [56], [28], [36].

The high hydraulic capacity and low resistance to embolism agrees with the high water availability inferred by the leaf physiognomy [28]. Since the fossil woods we studied here were collected in the meandering rivers facies and some of them have pelecypod perforations, it possible that, given their very low cavitation resistance values, the assemblage could be riparian. However, at this point, this is uncertain since the fossil woods were not collected in growth position, despite that several of them reached over 40 m (*Metcalfeoxylon* and *Javelinoxylon* xylotype 1). Whether the woods represent a riparian environment or not, extreme humidity of the Olmos Formation is independently confirmed by the leaf flora as it was collected in the flood plain-lagoon lithofacies [28].

Globally, it has been suggested that an increase in xylem and leaf hydraulic capacity during the mid to late Cretaceous [62], [63] likely influenced a significant amplification of angiosperm's forest biomass [64], and contributed to maximize carbon fixation [62] and expansion of tree size [64]. Indeed, our results indicate that the high conduction capacity of the woods from the Olmos paleoforest was necessary to sustain high leaf area [28] and high productivity predicted by paleoclimatic models [58], [59] for the region. High hydraulic capacity in the Olmos Formation paleoforest supports the current understanding of late cretaceous angiosperm hydraulic function, which proposes an increased conduction efficiency compared to early cretaceous short statured trees/shrubs [62], [63].

We suggest that the climate for the Olmos paleoforest, and likely other floras of the WINA, selected for an ecological strategy that maximized conductance and efficient carbon gain, and penalized high cavitation resistance because of its associated cost in hydraulic efficiency. As the probability of finding large pores in the pit membrane increases with vessel size [14], [20], [21], resistance to cavitation comes at expense of vessel size and conduction capacity. We suggest that the probability of drought in the Olmos paleoforest was low, otherwise the high vulnerability to cavitation of most species, together with a narrow range of ecological strategies along this functional aspect, would be an extremely risky strategy.

## Supporting Information

Table S1 **Vessel dimensions data for each of the samples/morphotypes.**
(XLSX)

Table S2 **Sample means for each functional trait.**
(XLSX)

Table S3 **Individual measurements of implosion resistance and estimated cavitation resistance.**
(XLSX)

Table S4 **Trait loadings in the three first PC axes.**
(XLSX)
